# Influence of Channel Surface with Ozone Annealing and UV Treatment on the Electrical Characteristics of Top-Gate InGaZnO Thin-Film Transistors

**DOI:** 10.3390/ma16186161

**Published:** 2023-09-11

**Authors:** Changyong Oh, Taehyeon Kim, Myeong Woo Ju, Min Young Kim, So Hee Park, Geon Hyeong Lee, Hyunwuk Kim, SeHoon Kim, Bo Sung Kim

**Affiliations:** 1 Department of Applied Physics, Korea University, Sejong 30019, Republic of Korea; steve481@korea.ac.kr (C.O.); skoks@korea.ac.kr (M.W.J.); noblegen@korea.ac.kr (M.Y.K.); ssohui225@korea.ac.kr (S.H.P.); startler@korea.ac.kr (G.H.L.); 2 E·ICT-Culture·Sports Track, Korea University, Sejong 30019, Republic of Korea; 3 Memory Diffusion Technology Team, Samsung Electronics, Pyeongtaek-si 17786, Republic of Korea; th0425.kim@samsung.com; 4 Display Development Division, ENF Technology Co., Ltd., Yongin-si 17084, Republic of Korea; caiskim@enftech.com (H.K.); kimsh@enftech.com (S.K.)

**Keywords:** InGaZnO (IGZO), top-gate thin-film transistors, stripper damage, ozone annealing, UV treatment

## Abstract

The effect of the channel interface of top-gate InGaZnO (IGZO) thin film transistors (TFTs) on the electrical properties caused by exposure to various wet chemicals such as deionized water, photoresist (PR), and strippers during the photolithography process was studied. Contrary to the good electrical characteristics of TFTs including a protective layer (PL) to avoid interface damage by wet chemical processes, TFTs without PL showed a conductive behavior with a negative threshold voltage shift, in which the ratio of Ga and Zn on the IGZO top surface reduced due to exposure to a stripper. In addition, the wet process in photolithography increased oxygen vacancy and oxygen impurity on the IGZO surface. The photo-patterning process increased donor-like defects in IGZO due to organic contamination on the IGZO surface by PR, making the TFT characteristics more conductive. The introduction of ozone (O_3_) annealing after photo-patterning and stripping of IGZO reduced the increased defect states on the surface of IGZO due to the wet process and effectively eliminated organic contamination by PR. In particular, by controlling surface oxygens on top of the IGZO surface excessively generated with O_3_ annealing using UV irradiation of 185 and 254 nm, IGZO TFTs with excellent current–voltage characteristics and reliability could be realized comparable to IGZO TFTs containing PL.

## 1. Introduction

Among several thin-film transistors (TFTs), including organic, amorphous Si, and low-temperature poly-Si TFT, oxide TFTs represented by InGaZnO (IGZO) have advantages such as high mobility, high current uniformity in a large area, low leakage current, and low subthreshold slope in terms of electrical characteristics. Therefore, oxide TFTs have been widely used as switching or driving devices for the backplane of active matrix displays, integrated circuits, and detectors [1,2,3,4,5,6,7]. Top-gate IGZO TFT is suitable as a TFT backplane structure of organic light emitting diode (OLED) that requires high-frequency operation, current uniformity, and electrical stability because it can establish a transistor structure similar to metal oxide semiconductor field-effect transistors (MOSFETs) with low parasitic capacitance and low interface trap states [8,9,10,11,12,13]. When fabricating top-gate TFTs, the top surface of the IGZO semiconductor is exposed to various chemicals such as photoresist (PR) and stripper during the photolithography process for forming an IGZO active island. In the top-gate structure, wet chemical processes involved in this photolithography can affect the characteristics of the TFT, because the IGZO top surface becomes the channel interface of the TFT later [14]. Several papers have reported TFT methodologies that perform photolithographic patterning after continuous deposition of gate dielectrics as a protective layer (PL) on IGZO to avoid the top surface being exposed to these chemicals [15,16,17]. The consecutive deposition of the gate dielectric PL on IGZO can prevent chemical damage on the channel surface, but since additional gate dielectrics must be deposited again after forming an active island pattern, the entire TFT fabrication process becomes complicated. Thus, it may not be favorable in terms of TFT productivity.

In this paper, we compared the differences in the electrical characteristics of IGZO TFTs containing PL and ones without PL under the same TFT fabrication conditions. In the case of IGZO TFTs without PL, the effect on electrical and optical properties from surface damage and contamination due to exposure to various chemicals such as PR, stripper, and rinsing water was investigated when IGZO was sputter-deposited and then active-patterned directly. Current–voltage (I–V) characteristics were correlated with changes in the IGZO surface. In addition, the IGZO surface treatment method and the defect healing mechanism were studied to improve the electrical properties of TFT related to chemical damage on the surface during the IGZO patterning process.

## 2. Materials and Methods

### 2.1. Fabrication of Top-Gate IGZO TFTs including PL (TFT-A Types)

On the top of a silicon wafer including the SiO_2_ layer, InSnO (ITO; 20 nm) was deposited and patterned to form source and drain (S/D) electrodes. IGZO (In:Ga:Zn = 1:1:1 atomic%, 30 nm) was then deposited with a radio frequency (RF)-magnetron sputtering system. An RF power source (13.56 MHz, 100 W) was used to deposit the IGZO layers. During deposition, the chamber working pressure was set to be 0.67 Pa with an argon (Ar): oxygen gas (O_2_) flow ratio of 45:5 standard cubic centimeters per minute at room temperature. Al_2_O_3_ (10 nm) as a protective layer was consecutively formed with thermal atomic layer deposition (ALD) at 250 °C to protect the top surface of IGZO. With respect to the deposition of the Al_2_O_3_ ALD protective layer, trimethyl aluminum (TMA) was used as an Al source, and ozone (O_3_) was used as a reactant. The thermal ALD process involved the following sequence: TMA injection (0.5 s)–Ar purge (10 s)–O_3_ injection (4 s)–Ar purge (10 s). The IGZO/Al_2_O_3_ double layer was patterned using wet etching with a 200:1 buffer oxide etchant. Al_2_O_3_ (100 nm) as a gate insulator was grown with PEALD under an O_2_ plasma power of 100 W at 250 °C. The PEALD process was performed with the following sequence: TMA injection (0.5 s)–Ar purge (3 s)–O_2_ injection (1 s)–O_2_ plasma (0.5 s)–Ar purge (2 s). After S/D contact holes were formed on the Al_2_O_3_ dielectric layer (100 nm) using photolithography, a Mo (50 nm) gate electrode was sputter-deposited and patterned to afford the IGZO TFTs including PL (TFT-A). The TFT-A devices were then annealed at 350 °C in the air for 1 h.

### 2.2. Fabrication of Top-Gate IGZO TFTs without PL (TFT-B Types)

The IGZO TFTs without PL (TFT-B) were prepared with the same method until the IGZO deposition step. IGZO films were photo-patterned and wet-etched with a buffered oxide etchant (200:1). Al_2_O_3_ as a gate insulator layer was formed using a sequential deposition process of thermal ALD (10 nm) and PEALD (100 nm) at 250 °C. The thermal ALD and PEALD processes for Al_2_O_3_ deposition were performed in the same sequences with TFT-A. After S/D contact holes were formed on the Al_2_O_3_ dielectric layer (110 nm) using photolithography, a Mo (50 nm) gate electrode was sputter-deposited and patterned to afford the IGZO TFTs without PL (TFT-B). The TFT-B devices were then annealed at 350 °C in the air for 1 h.

### 2.3. Electrical and Optical Measurements of IGZO TFTs

Electrical characteristics of stretchable IGZO TFTs were measured using a semiconductor device analyzer (B1500A, Agilent, Santa Rosa, CA, USA) at ambient air temperature in the dark. The current–voltage (I–V) characteristics of TFTs were measured with a gate voltage step of 0.5 V and constant drain voltage (V_d_) of 1 V. The thicknesses of the gate and drain metal electrodes were measured with a surface profiler (D-600, KLA Tencor, Milpitas, CA, USA). The thicknesses of Al_2_O_3_ and IGZO were measured with an ellipsometer (SEMG-1000, Nanoview, Seoul, Republic of Korea). Atomic force microscope (AFM; Park systems, Suwon, Republic of Korea) analysis was performed to confirm whether wet chemical changes the morphology of the IGZO surface. Chemical states of oxygen atoms in IGZO films were examined using X-ray photoelectron spectroscopy (XPS; K-Alpha^+^, Thermo Fisher Scientific, Waltham, MA, USA). Spectroscopic ellipsometry analysis of IGZO films was also conducted with a Nanoview ellipsometer. Work function and valence band maximum of IGZO films were measured by UV photoelectron spectroscopy (UPS; Axis-Supra, Kratos, Manchester, UK) with incident photon energy (E_incident_) of 21.2 eV.

## 3. Results and Discussion

**Figure 1:**
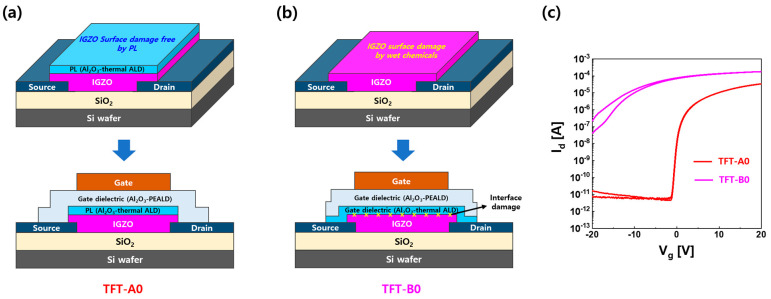
Comparison between the process architectures of (**a**) a top-gate IGZO TFT with IGZO and Al_2_O_3_ PL depositions followed by photolithography for patterning an active island (TFT-A0) and (**b**) a top-gate IGZO TFT with IGZO deposition and photolithography followed by Al_2_O_3_ deposition (TFT-B0). (**c**) I–V characteristics of TFT-A0 and TFT-B0.

Figure 1a,b show a comparison of the process architectures of top-gate IGZO TFTs including PL and ones without PL, respectively. As shown in Figure 1a, for the fabrication of IGZO TFTs including PL (TFT-A0), an IGZO layer was deposited on ITO S/D using RF-sputtering with an oxygen partial pressure of 5%, and Al_2_O_3_ as a protective layer on the IGZO top surface was consecutively formed with thermal ALD using TMA as an Al source and an O_3_ reactant at 250 °C. TFT-A0 was produced by patterning the IGZO/PL active island, which prevented the IGZO channel interface from being exposed to wet chemicals during the active patterning process. On the other hand, as shown in Figure 1b, sputter-deposition of the IGZO layer was carried out under the same conditions as that of TFT-A0, but TFT-B0 was produced by patterning an IGZO active island without PL so that the IGZO top surface was exposed to various wet processes such as PR coating, stripping, and rinsing with deionized water (DI water) during the active photolithography process. Figure 1c shows the I–V characteristics of TFT-A0 and TFT-B0. TFT-A0 showed electrical properties of a typical IGZO TFT with field-effect mobility (μ_FE_) of 5.6 cm^2^V^−1^s^−1^, threshold voltage (V_th_) of 0.13 V, on/off current ratio of 7.1 × 10^6^, and low subthreshold slope of 0.25 V/dec. In contrast, TFT-B0 showed completely different I–V characteristics, exhibiting conductive behavior (μ_FE_ of 14.6 cm^2^V^−1^s^−1^, V_th_ below −20 V) with high drain current. It could be expected that wet chemicals, such as PR (GXR601, AZ), stripper (MR-STR-100, Mattech Resource), and deionized water, used during the photolithography process for IGZO patterning affected the IGZO surface.

**Figure 2:**
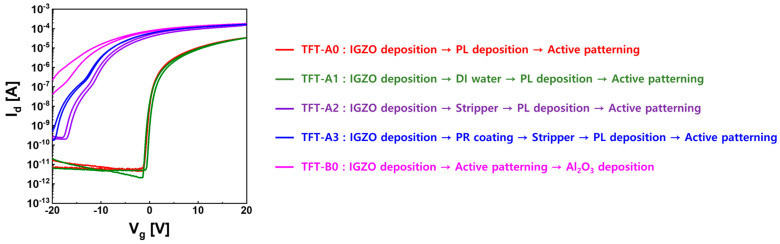
I–V characteristics of top-gate IGZO TFTs according to various wet chemical treatments applied to the IGZO surface.

To determine the cause of the conductive property of TFT-B0, when fabricating TFT devices with PL, wet chemical treatments such as simple DI water rinse (TFT-A1), stripper (TFT-A2), PR coating, and stripper (TFT-A3) on the IGZO surface, followed by PL deposition were carried out to afford TFT-A type devices, and then their electrical characteristics were compared. The reason for comparing these different wet treatment methods was to find out which chemical treatment dominantly affected the conductive property of TFT-B0. As shown in Figure 2, in the case of only using DI water cleaning of the IGZO surface (TFT-A1), there was no significant difference in electrical characteristics compared to TFT-A0. The stripper treatment on the IGZO surface (TFT-A2) showed severe depletion mode transistor operation along with a negative shift in V_th_ and significantly increased drain current. TFT-A3, which involved PR coating and a strip process on the IGZO surface, showed a more negative V_th_ shift than TFT-A2, representing similar results to TFT-B0. Therefore, chemicals such as PR and stripper affect TFT characteristics. In particular, a stripper was found to provide the main conductive properties of IGZO TFTs.

**Figure 3:**
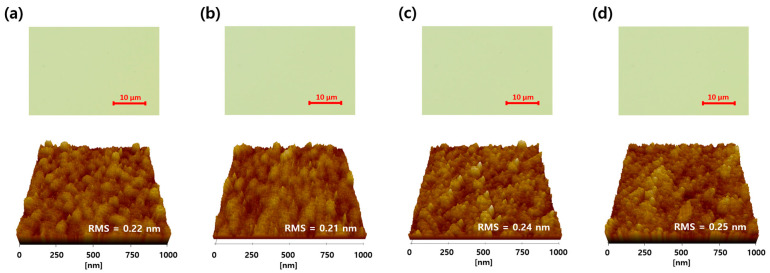
Optical microscope images (top) and AFM images (bottom) of IGZO thin films: (**a**) as-deposited, (**b**) DI-water rinsed, (**c**) after strip treatment, and (**d**) after PR coating and strip treatment.

**Figure 4:**
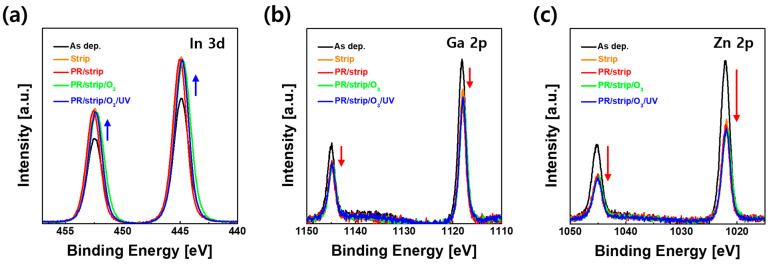
XPS spectra of (**a**) In 3d, (**b**) Ga 2p, and (**c**) Zn 2p in the top surface of IGZO films according to various surface treatments.

IGZO films with various treatments were observed with an optical microscope to determine whether the wet chemicals caused surface contaminations. AFM analysis was also performed to identify whether wet chemicals changed the morphology of the IGZO surface. As shown in Figure 3, both IGZO top surfaces (top images) obtained with the microscope and surface morphologies obtained with AFM (bottom images) showed little change after wet chemical treatments compared to that of the as-deposited IGZO film. Meanwhile, an XPS analysis was conducted to determine the cause of the conductive property of IGZO TFT with the stripper. Table S1 shows the changes in atomic % of the metals and oxygen composition in the IGZO bulk and surface using the stripper only and PR/stripper treatments. As shown in Figure S1, in the IGZO bulk layer, each peak area of In 3d, Ga 2p, and Zn 2p showed little change regardless of the wet chemical treatment used, such as stripper and PR/stripper, compared to as-deposited IGZO film. These were also consistent with the results of the XPS O 1s spectra of the IGZO bulk region, as shown in Figure S2a–c. However, changes in metal composition were clearly observed on the IGZO surface; in particular, the ratios of Ga (4.6 to 3.1%) and Zn (6.8 to 3.7%) significantly decreased after the PR coating and stripping treatment, as shown in Figure 4. This indicated that some of the Ga and Zn atoms on the surface were dissolved by the PR solvent and stripper solution.

**Figure 5:**
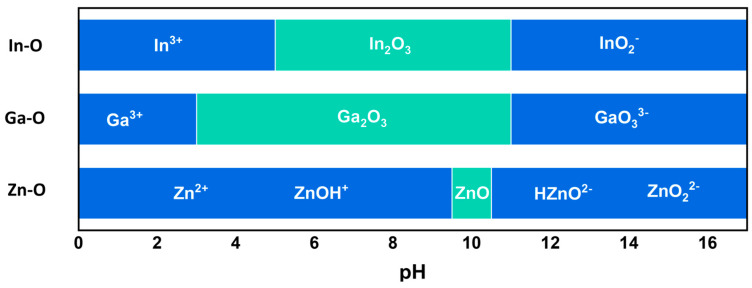
Theoretical Pourbaix diagram for phase transition of IGZO.

We investigated the solubility of oxide compounds related to In, Ga, and Zn metals with respect to pH to understand the change in the metal composition in IGZO with the stripper. Figure 5 shows the theoretical Pourbaix diagram for the phase transition of IGZO, where the insoluble pH regions of In_2_O_3_, Ga_2_O_3_, and ZnO exhibit 5–11, 3–11, and 9.5–10.5, respectively [18,19]. The metal oxide anion forms of these three components are preferred near pH 11. Because the potential difference (i.e., electromotive force) in these reactions has a positive value when the pH is greater than 11, in the following Equations (1)–(3), it is thermodynamically preferred that these chemical reactions occur in the forward direction. Since the MR-STR-100 stripper used here exhibited a pH of 11, it could be assumed that the IGZO might be affected by the stripper.

(1) In_2_O_3_ + 2 OH^−^ ⇌ 2 InO_2_^−^ + H_2_O

(2) Ga_2_O_3_ + 2 OH^−^ ⇌ 2 GaO_2_^3−^ + H_2_O

(3) ZnO + 2 OH^−^ ⇌ ZnO_2_^2−^ + H_2_O

**Figure 6:**
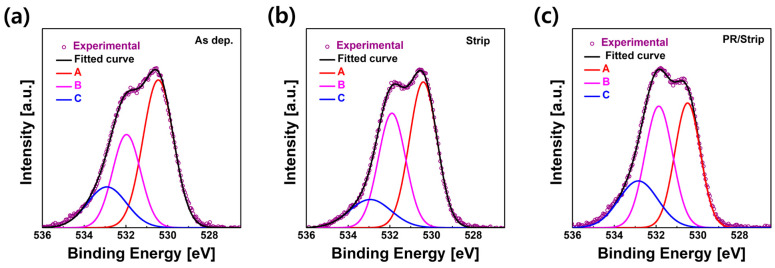
XPS O 1s spectra of the top surface of IGZO films (**a**) as-deposited, (**b**) after stripper treatment, and (**c**) after PR coating and stripper treatment.

From the XPS quantitative analysis of the IGZO films, it was found that the relative ratio of In atoms on the IGZO surface increased with the stripper treatment. This could increase conduction band dispersion due to the decrease in the effective mass of electrons in IGZO and decrease the activation energy due to an increase in the V_O_ defect in IGZO, resulting in an increase in the concentration of electrons on the surface [20,21,22,23]. The oxygen-related defects in the IGZO surface according to the wet chemical processes were also investigated using an XPS O 1s analysis. Figure 6 shows the XPS O 1s spectra of the surfaces of the as-deposited IGZO film, stripper-treated IGZO film, and PR/stripper-treated IGZO film, which refer to the chemical environment of the IGZO surface of TFT-A0, A2, and A3, respectively (an XPS analysis of IGZO related to TFT-A1 was skipped since the electrical property of TFT-A1 was the same as that of TFT-A0). The O 1s peaks on the IGZO surface for each sample were deconvoluted into three Gaussian subpeaks at 530.3, 531.7, and 532.9 eV. The peak A centered at 530.3 eV is associated with oxygen bound to fully coordinated metals. The peak B at 531.7 eV in the middle corresponds to oxygen atoms of oxygen-deficient regions associated with oxygen vacancy (V_O_). The high binding energy peak (peak C) located at 532.9 eV indicates impurity-related oxygen atoms such as O interstitials, hydroxyl (O-H) groups, and C-O bonds [24,25,26,27,28]. 

Using the area ratio of these individual peaks, we compared the relative amounts of oxygens associated with fully coordinated metals, V_O_, and O-impurity in IGZO thin films (Table S2). In the case of the IGZO surface treated with the stripper, the area ratio of peaks A, B, and C decreased from 51.8% to 48.4%, increased from 30.0% to 37.5%, and increased from 18.3% to 14.1%, respectively. These results might be due to an increase in V_O_ (i.e., an increase in the In ratio) in the IGZO surface due to the stripper, which was related to the I–V characteristics of TFT-A2. In the case of stripping after the PR process on the IGZO surface, the areas of peak B and peak C were 40.2% and 21.8%, respectively. These peaks B and C increased more than those for only the stripper treatment. Therefore, it was found that V_O_ and O-impurity significantly increased, and fully saturated metal oxide (M-O) bonds decreased in the PR/stripper-treated IGZO film. This could lead to an increase in carrier concentration in IGZO and cause conducting phenomena such as TFT-A3. In particular, the increase in the area of peak C could be due to the contribution of impurities such as C-O bonds originating from PR. As shown in Figure S3a, in the C1s spectra of XPS, a significant increase in C-O and C-C peak intensity was observed on the IGZO surface treated with PR/stripper. As C-O bond-related impurities form donor-like defects, they might have increased carrier concentration in IGZO, resulting in conducting effects such as TFT-A3 and TFT-B0 [14]. 

In addition, humps occurred in the I–V curves for TFT-A2 and A3. The PR/strip treatment on IGZO resulted in an increase in Vo, In ratio, and carbon impurity. Various cases of hump phenomena occurring in IGZO TFTs have been reported, including studies on back-channel charge trapping of positively charged species (V_O_^2+^, Zn^2+^, Na^+^) in IGZO with DC gate bias, channel edge trapping of H^+^ in hydrogen annealing, path generation of oxygen vacancies created in IGZO by the bending stress of TFT, and back-channel trapping of holes in electron/hole pairs generated by positive bias with bending stress [29,30,31,32]. Here, these defects could increase free carrier and shallow donor states in the subgap of IGZO. According to the subgap DOS simulation of IGZO TFT, it is known that increased shallow donor states (N_GD_) in IGZO could cause a hump with a negative V_th_ shift [33].

In the IGZO patterning process, surface treatments on IGZO were studied to improve the electrical properties of TFT by restoring the chemical damage on the surface and removing contamination. O_3_ treatment is known as an effective way to remove organic contamination from an IGZO surface [34,35,36,37,38]. In addition, the annealing process containing O_3_ gas can lower V_O_ on an IGZO surface exposed to a stripper and increase the fully coordinated M-O bond, controlling the carrier concentration [39]. Here, in the process of IGZO patterning, O_3_ annealing was introduced to remove organic contamination by PR and control the increased V_O_ on the IGZO caused by the stripper. Figure S4 shows typical I_d_–V_g_ and I_g_–V_g_ characteristics of top-gate IGZO TFTs with different treatments on IGZO surfaces. The gate leakage current increased up to the level of 10^−10^ A with the stripping process before PL deposition. However, using O_3_ annealing or O_3_ annealing/UV treatment, the gate leakage was greatly reduced to the level of 10^−12^ A. This might be due to the cleaning effect of the organic matter remaining on the surface of IGZO. 

**Figure 7:**
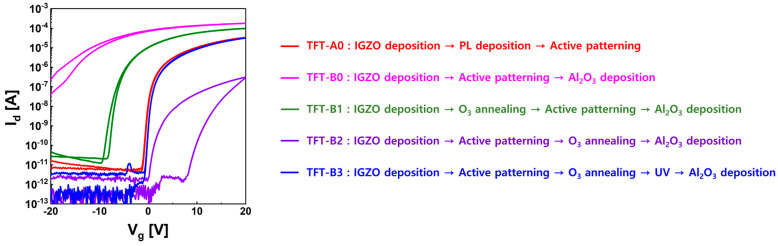
I–V characteristics of top-gate IGZO TFTs according to O_3_ annealing before and after IGZO patterning.

Figure 7 shows the electrical characteristics of IGZO TFTs when O_3_ annealing was used at 350 °C for 30 min before and after IGZO patterning. The I–V curve for TFT-B1 is the result of using O_3_ annealing before IGZO patterning, and that for TFT-B2 is the result of O_3_ annealing after IGZO patterning. TFT-B1 showed improved transistor properties with O_3_ annealing, but it still operated in the depletion mode. This may have been because the IGZO surface damage on TFT-B1 still existed with the consecutive wet process after O_3_ annealing, although V_O_-related defects in IGZO were passivated with the oxygen source that came from O_3_ annealing. On the other hand, TFT-B2 was excessively deactivated along with hysteresis in the transfer curve with O_3_ annealing after the IGZO-patterning process. This might be attributed to an increase in oxygen interstitials (O_i_) and a decrease in carrier concentration due to the contribution of O atoms to the V_O_ sites of IGZO by the excess oxygen source. Since the oxygen interstitials in IGZO act as acceptor states, they could play the role of carrier traps in positive gate bias and cause clockwise hysteresis [40]. O_3_ annealing could not only eliminate organic impurity contaminated on IGZO by the PR process but also effectively control carrier concentrations due to V_O_ and In metal on the IGZO surface. However, the extra O_i_ remaining on the IGZO surface when using O_3_ annealing played a role in lowering the performance of TFT. As a method to control excess O_i_ on the IGZO surface and activate semiconductor properties, deep UV with wavelengths of 185 nm and 254 nm was irradiated on the IGZO surface after O_3_ annealing. We used a low-pressure mercury lamp (G12T5VH/4P, Han Sung Ultraviolet Co., Seongnam, Republic of Korea) with a power of 80 W and a luminous intensity of 20 mWcm^−2^ as a UV light source. Figure S5 shows the spectrum of the UV lamp. As shown in Figure 7, TFT-B3 irradiated with UV after O_3_ annealing showed excellent I–V characteristics similar to TFT-A0. 

**Figure 8:**
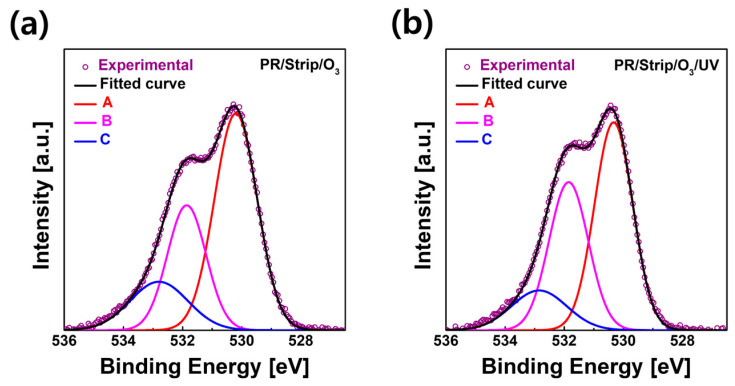
XPS O 1s spectra of the top surface of IGZO films: (**a**) O_3_-annealed and (**b**) O_3_/UV-treated.

**Figure 9:**
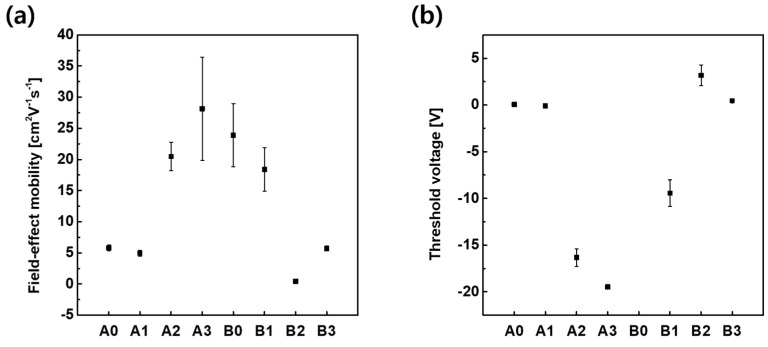
Average and standard deviation for (**a**) field-effect mobilities and (**b**) threshold voltages of top-gate IGZO TFTs with different treatments on IGZO surfaces.

Figure 8a,b show the O 1s XPS spectrum of the IGZO film treated with O_3_ annealing after photo-patterning and the IGZO film treated with O_3_ annealing/UV after photo-patterning (PR/strip), respectively. PR/strip/O_3_-annealed IGZO (Figure 8a) obviously decreased O-impurity (16.6%) and V_O_ (28.6%) and significantly increased M-O (54.8%) on the surface compared with the PR/strip-treated IGZO (Figure 6c). On the other hand, in the PR/strip/O_3_ annealing/UV-treated IGZO, V_O_ (36.4%) increased slightly again, but O-impurity (13.8%) decreased further (Figure 8b). According to the XPS O 1s spectrum, it appears that UV treatment not only regulated V_O_ production but also contributed to the removal of O_i_-related defects. In other words, the strong energy of deep UV was expected to reduce O_i_-related defects by dissociating the peroxide (O-O) bonds remaining on the IGZO surface using O_3_ annealing [41]. The excellent electrical properties of TFT-B3 would be attributed to the removal of excess oxygen (i.e., O_i_-related impurity) weakly bound to the IGZO surface due to UV irradiation in addition to the controlled V_O_. Figure 9a,b shows the average and standard deviation for the field effect mobility and threshold voltage of three TFTs for each case of surface treatment, respectively. Field-effect mobility was extracted in the saturation region, and V_th_ was extrapolated from the $\sqrt{I_{d}}$–V_g_ curve. TFT-A0, A1, and B3 had generally similar mobility and V_th_ and showed very low deviation. The average mobility for TFT-A0, A1, and B3 was 5.9 ± 0.48, 5.0 ± 0.48, and 5.7 ± 0.40 cm^2^V^−1^S^−1^, respectively.

**Figure 10:**
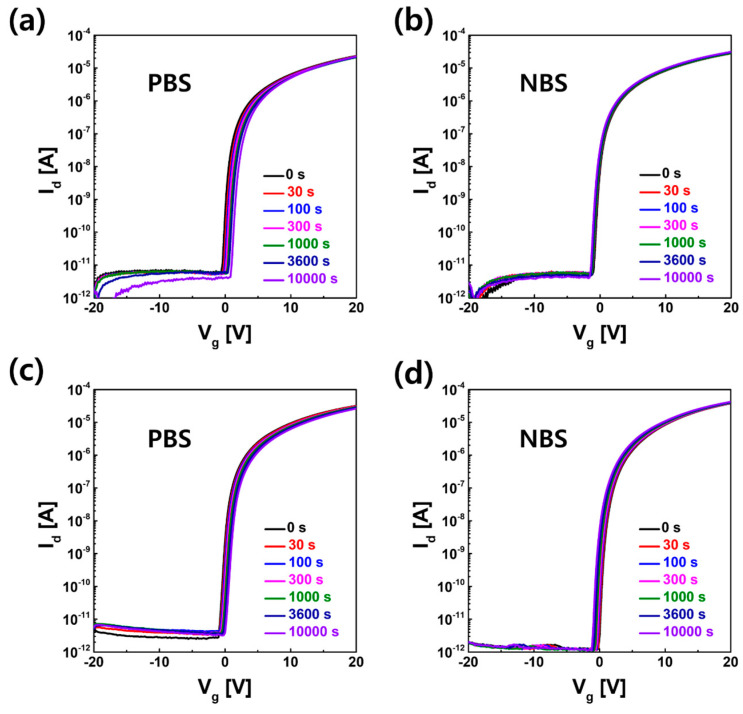
I–V characteristics for TFT-A0 under (**a**) positive gate bias stress (PBS) and (**b**) negative gate bias stress (NBS) and TFT-B3 under (**c**) PBS and (**d**) NBS.

The electrical reliability against positive gate bias stress (PBS) and negative gate bias stress (NBS) was compared between TFT-A0 and TFT-B3, which showed excellent electrical properties. As shown in Figure 10, TFT-A0 without wet chemical damage owing to PL exhibited a V_th_ shift of +1.5 V under the PBS condition (V_g_ = +20 V, V_d_ = +0.1 V, 10,000 s) and −0.4 V under the NBS condition (V_g_ = −20 V, V_d_ = +10 V, 10,000 s), respectively. TFT-B3 treated with PR/strip/O_3_ annealing/UV showed a V_th_ shift of +1.0 V under the PBS condition and −0.9 V under the NBS condition, respectively. This meant that the surface of IGZO damaged after exposure to the PR/stripper treatment was cured with the O_3_ annealing/UV treatment by reducing the interface trap states. Thus, TFT-B3 showed sufficiently good reliability comparable to that of TFT-A0.

**Figure 11:**
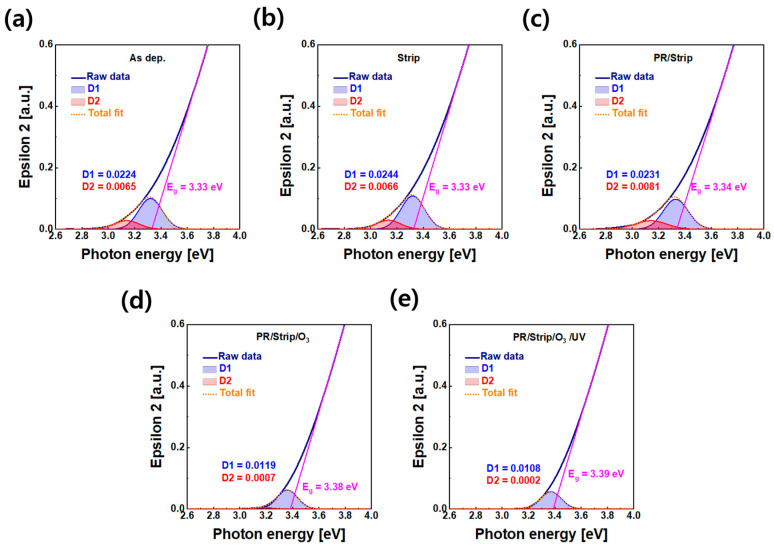
Spectroscopic ellipsometry analysis of IGZO films: (**a**) as-deposited, (**b**) after strip treatment, (**c**) after PR/strip treatment, (**d**) after O_3_ annealing, and (**e**) after O_3_ annealing/UV.

Figure 11 shows the spectroscopic ellipsometry analysis of the subgap states below the conduction band according to different process treatments, including the as-deposited, stripper, PR/stripper, O_3_ annealing, and O_3_/UV, on IGZO films. The reliability of TFTs can be interpreted by comparing the imaginary dielectric function spectra of these IGZO films. Based on the Gaussian model fitting of the ellipsometry spectrum, the band edge states of IGZO were deconvoluted into two subgap states (D1 and D2). Each peak of two subgap states was located below 0.02 eV and 0.2 eV from the conduction band edge, respectively [42,43]. Shallow band edge states (D1) act as donor states that emit free electrons, resulting in TFT-A2 and TFT-A3. Deep band edge states (D2) act as charge trapping sites or induce charge scattering and are associated with PBS instability of TFTs [44,45]. It was confirmed that the area of D1 and D2 was outstandingly reduced with O_3_ annealing. Here, D1 might have originated from V_O_, In ion, and carbon impurity during the PR/strip process, which could be reduced with O_3_-annealing, suppressing the conductive behavior of TFT-A2 and TFT-A3. It was also found that there was little difference in the region of band edge states in the optical bandgap between O_3_ annealing and the O_3_ annealing/UV treatment, which was consistent with the results of the XPS O 1s spectrum in the IGZO bulk region (Figure S2d,e). This was thought to be because UV irradiation did not significantly affect the entire IGZO film but only affected the IGZO surface. This limited effect on the IGZO surface might be due to room-temperature UV treatment without thermal diffusion or high energy ion penetration.

**Figure 12:**
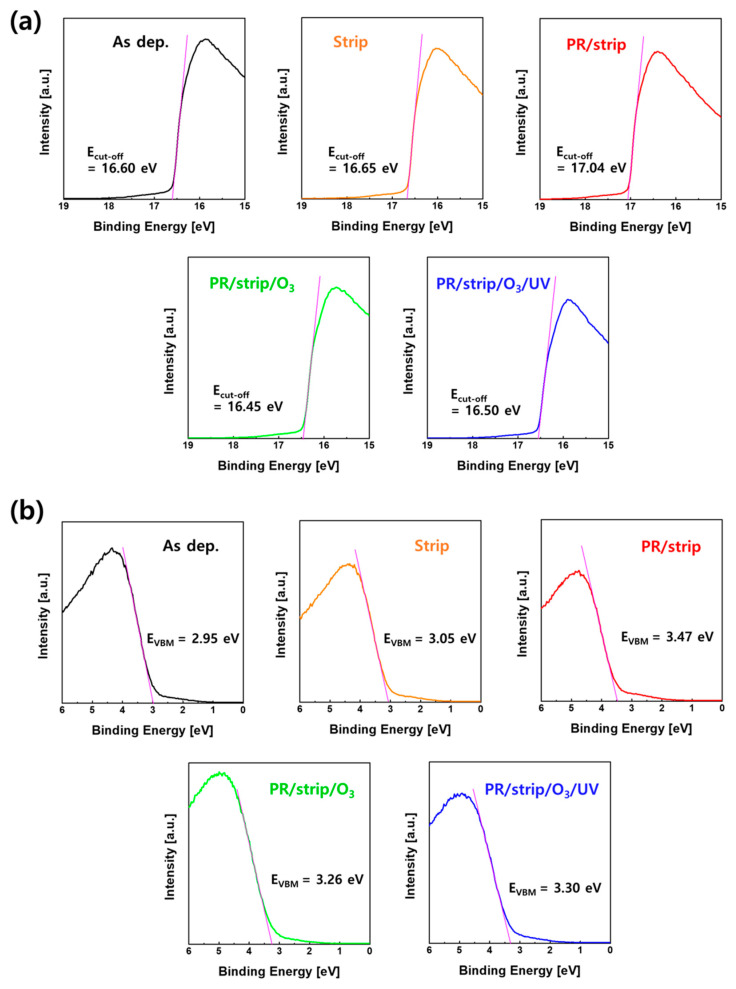
(**a**) Secondary electron cut-off energy (E_cut-off_) and (**b**) valence band maximum (E_V_) in UV photoelectron spectroscopy of IGZO films with different treatments on the IGZO surface.

**Figure 13:**
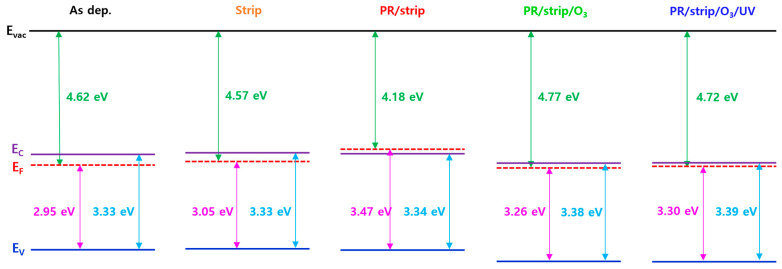
Energy band diagrams extracted from ellipsometry and UPS analysis of IGZO films with different treatments on IGZO surfaces.

A UPS analysis was conducted to further understand the correlation between the different electrical properties of IGZO TFTs due to the various surface treatments and the energy state of valence electrons participating in chemical bonding in IGZO. UPS is a useful analysis technology for identifying IGZO valence band states like XPS by finely grasping the electron emission energy in the valence band [46,47]. Figure 12a,b shows secondary electron cut-off energy (E_cut-off_) and valence band maximum (E_V_) in the UV photoelectron spectroscopy of IGZO films with different treatments on the IGZO surface. The work function of IGZO films [Φ = hv − (E_F_ − E_cut-off_)] and the energy of E_F_-E_V_ were calculated using E_cut-off_ and E_V_, respectively. As shown in Figure 13, energy band diagrams were extracted from ellipsometry spectroscopy and the UPS analysis of IGZO films with different treatments on the IGZO surface. The fact that E_F_ exists above E_C_ in the energy band diagram of IGZO with the PR/strip treatment condition might explain why TFT-B0 shows conductive behavior. This increase in E_F_ could be attributed to the effects of carbon contamination in the C 1s spectra of XPS, as shown in Figure S3a. In addition, E_F_ would be significantly lowered due to carbon removal with O_3_ annealing after PR/strip treatment, resulting in TFT-B2. However, there was no special change in the Na 1s spectra of the top surface of IGZO films according to different treatments, as shown in Figure S3b. O_3_ annealing obviously reduces the carrier concentration in IGZO to deactivate TFT. Nevertheless, the influence of the PR/stripper treatment on the IGZO surface was expected to be strong enough to increase carrier concentration again due to the increase of In ratio and oxygen vacancy as well as carbon contamination on the IGZO surface. This might have caused TFT-B1 to operate in a considerable depletion mode. As a result, the analyses of XPS, UPS, and ellipsometry spectroscopy confirmed that O_3_ annealing/UV irradiation is a good surface treatment method that can improve the properties and reliability of top-gate IGZO TFTs without PL (i.e., TFT-B3) by removing organic contamination and repairing O-related defects on the IGZO surface without affecting IGZO bulk.

## 4. Conclusions

In terms of the fabrication of top-gate IGZO TFTs, the top surface of the IGZO semiconductor eventually becomes the front channel interface of the TFT, so it is crucial to minimize surface damage by wet chemicals during the photolithography process and to effectively manage the channel interface. The effect of the channel surface damage of IGZO semiconductors due to exposure to various wet chemicals such as deionized water, photoresist, and stripper was examined on the electrical properties of the IGZO TFTs. Compared with IGZO including PL without interface damage, IGZO without PL had significantly reduced ratios of Ga and Zn (increased In ratio) in the top surface due to exposure to the stripper. The XPS O 1s analysis showed that photo-patterning and the stripping process increased the V_O_ and O-impurity of the IGZO surface. This would have resulted in an increase in free carriers in IGZO and contributed to negative V_th_ shift and conductive behavior in the TFT characteristics. The photo-patterning process might have also increased donor-like defects in IGZO due to organic contamination on the IGZO surface by PR, making the TFT characteristics more conductive. The introduction of O_3_ annealing after photo-patterning and stripping of IGZO enabled a reduction in the increased V_O_ states on the surface of IGZO due to the wet process and effectively eliminated organic contamination by PR. In particular, by controlling excessive surface oxygen on top of the IGZO surface excessively generated using O_3_ annealing with deep UV irradiation, IGZO TFTs with excellent I–V characteristics and reliability could be realized comparable to IGZO TFTs containing a protective layer.

## Data Availability

Not applicable.

## Supplementary Materials

The following supporting information can be downloaded at: https://www.mdpi.com/article/10.3390/ma16186161/s1, Figure S1: XPS spectra of (a) In 3d, (b) Ga 2p, and (c) Zn 2p in bulk region of IGZO films according to various surface treatments; Figure S2: XPS O 1s spectra in bulk region of IGZO films (a) as-deposited, (b) after stripper treatment, (c) after PR-coated and striped, (d) after O3 annealing, and (e) after O3 annealing/UV; Figure S3: XPS spectra of (a) C 1s and (b) Na 1s of top surface of IGZO films with different treatments; Figure S4: I_d_–V_g_ and I_g_–V_g_ characteristics of top gate IGZO TFTs with different treatments on IGZO surfaces; Figure S5: The spectrum of a low-pressure mercury lamp: It shows two peaks (185 and 254 nm), the major peak is 254 nm; Table S1: The changes in the composition of In, Ga, Zn, and O atoms of the IGZO bulk and surface by various chemical treatments on top of IGZO layer; Table S2: Relative amounts of O 1s associated with fully coordinated metals, VO, and O-impurity in IGZO thin films.
