# Supplementary material for: Influence of Channel Surface with Ozone Annealing and UV Treatment on the Electrical Characteristics of Top-Gate InGaZnO Thin-Film Transistors
Source: Materials (Basel). 2023 Sep 11;16(18):6161. doi: 10.3390/ma16186161 (PMC10532450; doi:10.3390/ma16186161)
Supplement: Supplementary file 1 [file materials-16-06161-s001.zip › materials-2585123-supplementary.pdf]

Supplementary Information

Influence of channel surface by ozone annealing and UV treatment on the electrical characteristics of top gate InGaZnO thin-film transistors

Changyong Oh[1,2], Taehyeon Kim[3], Myeong Woo Ju[1,2], Min Young Kim[1], So Hee Park[1], Geon Hyeong Lee[1], Hyunwuk Kim[4], SeHoon Kim[4] and Bo Sung Kim[1,2,*]

[1] Department of Applied Physics, Korea University, Sejong, 30019, Republic of Korea

[2] E·ICT-Culture·Sports Track, Korea University, Sejong, 30019, Republic of Korea

[3] Memory Diffusion Technology Team, Samsung Electronics, Pyeongtaek-si, Gyeonggi-do, 17786, Republic of Korea

[4] Display Development Division, ENF Technology Co., Ltd., Yongin-si, Gyeonggi-do, 17084, Republic of Korea

Table S1. The changes in the composition of In, Ga, Zn, and O atoms of the IGZO bulk and surface by various chemical treatments on top of IGZO layer.

		In [at%]	Ga [at%]	Zn [at%]	O [at%]
Bulk	As dep.	12.7	7.3	9.9	70.1
	Strip	12.9	7.3	9.9	70.0
	PR/strip	12.9	7.1	10.2	69.7
	O_3 anneal	12.9	7.2	10.1	69.7
	O_3 anneal/UV	12.9	7.2	10.2	69.7
Surface	As dep.	12.8	4.6	6.8	75.7
	Strip	16.3	3.4	3.8	76.5
	PR/strip	16.2	3.1	3.7	77.0
	O_3 anneal	16.0	3.1	3.4	77.4
	O_3 anneal/UV	16.1	3.0	3.6	77.2

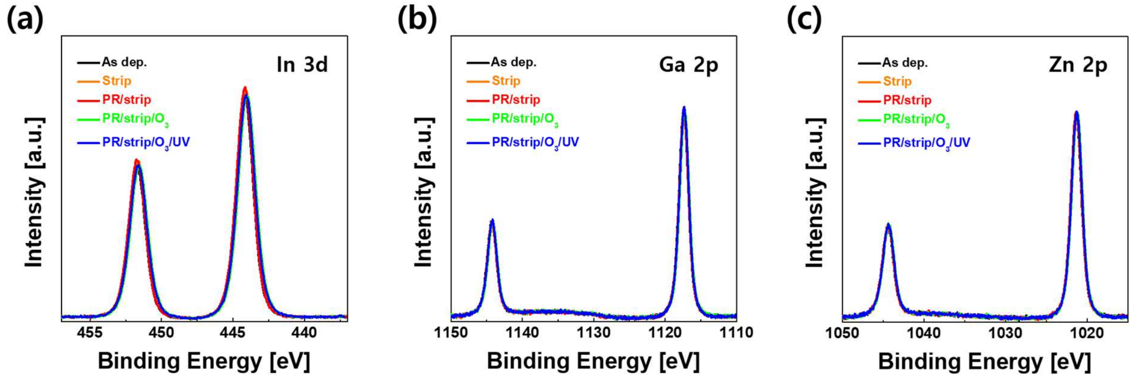

Figure S1. XPS spectra of (a) In 3d, (b) Ga 2p, and (c) Zn 2p in bulk region of IGZO films according to various surface treatments.

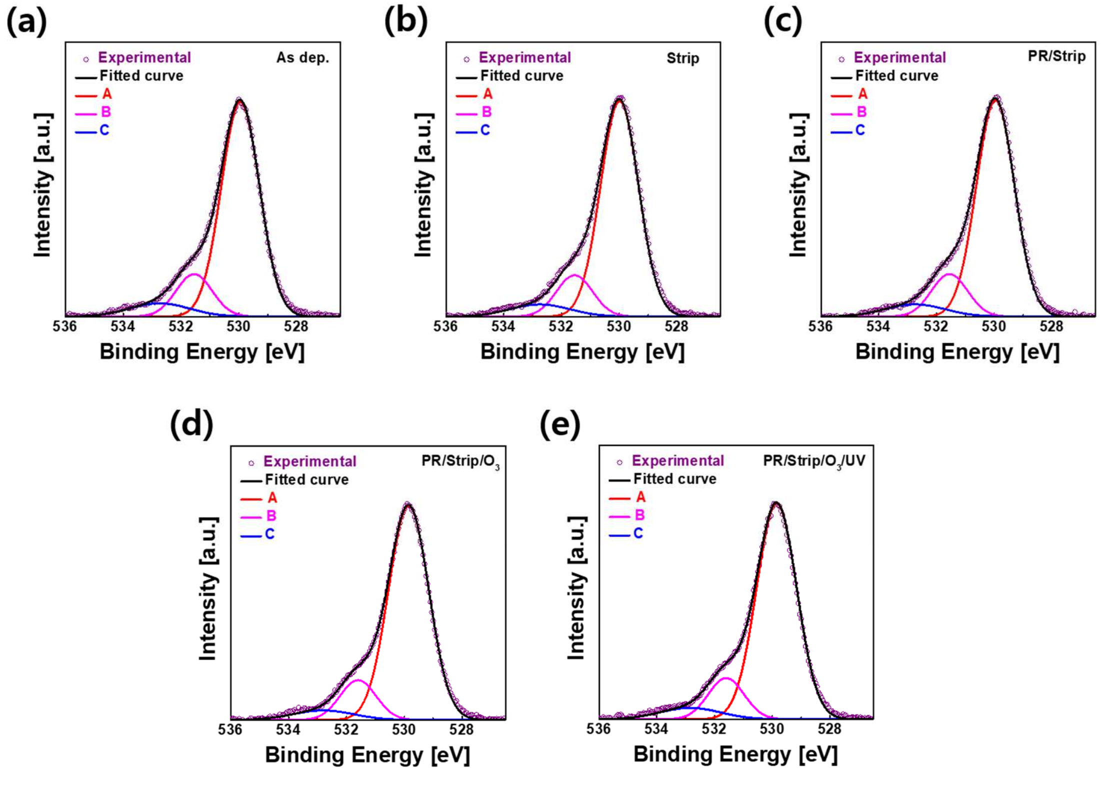

Figure S2. XPS O 1s spectra in bulk region of IGZO films (a) as-deposited, (b) after stripper treatment, (c) after PR-coated and striped, (d) after O_3 annealing, and (e) after O_3 annealing/UV.

Table S2. Relative amounts of O 1s associated with fully coordinated metals, V_O, and O-impurity in IGZO thin films.

		530.3 ± 0.2 eV<br>peak A [%]	531.7 ± 0.2 eV<br>peak B [%]	522.9 ± 0.2 eV<br>peak C [%]
Surface	As dep.	51.8%	30.0%	18.3%
	Strip	48.4%	37.5%	14.1%
	PR/strip	38.0%	40.2%	21.8%
	O_3 anneal	54.8%	28.6%	16.6%
	O_3 anneal/UV	49.8%	36.4%	13.8%
Bulk	As dep.	78.0%	14.5%	7.5%
	Strip	78.7%	14.3%	7.0%
	PR/strip	78.4%	14.6%	7.0%
	O_3 anneal	81.2%	13.4%	5.4%
	O_3 anneal/UV	80.9%	13.6%	5.5%

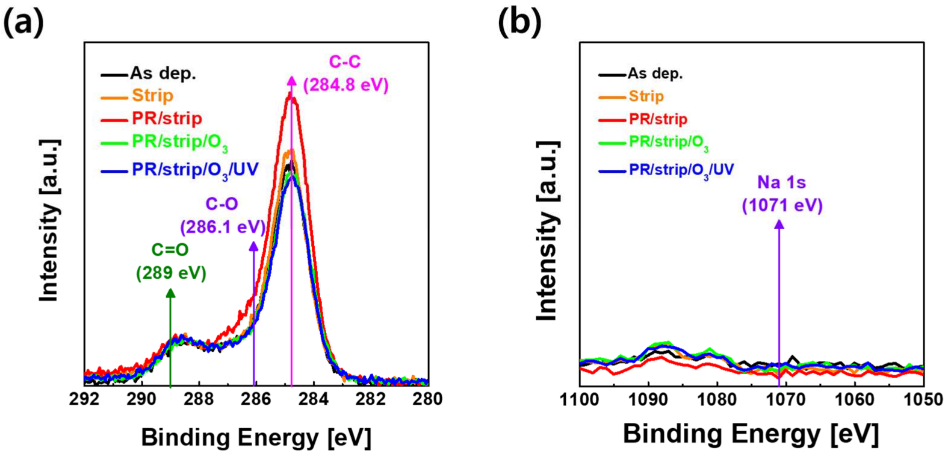

Figure S3. XPS spectra of (a) C 1s and (b) Na 1s of top surface of IGZO films with different treatments.

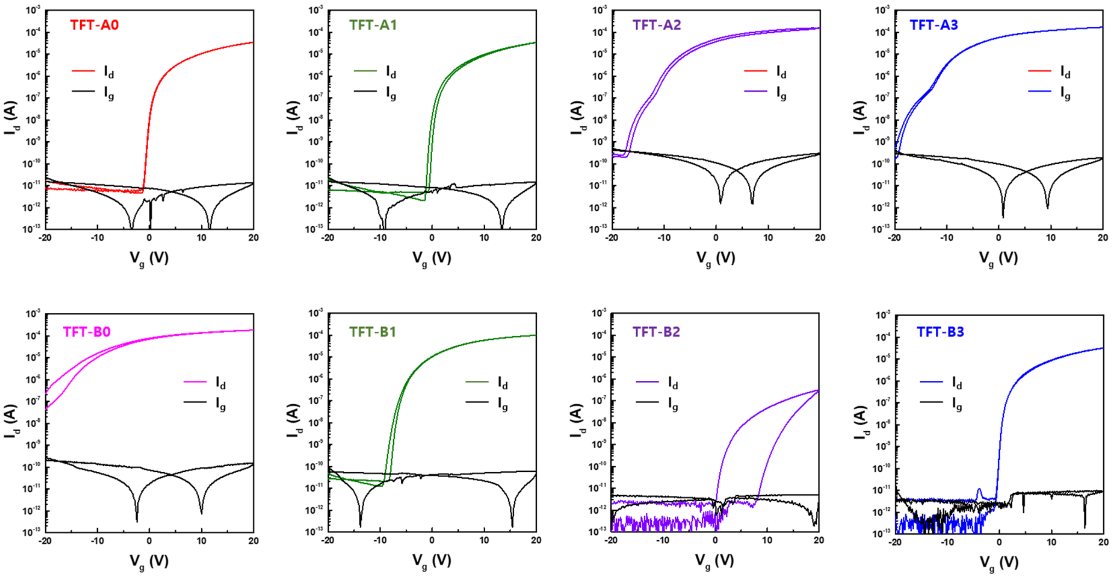

Figure S4. I_d-V_g and I_g-V_g characteristics of top gate IGZO TFTs with different treatments on IGZO surfaces.

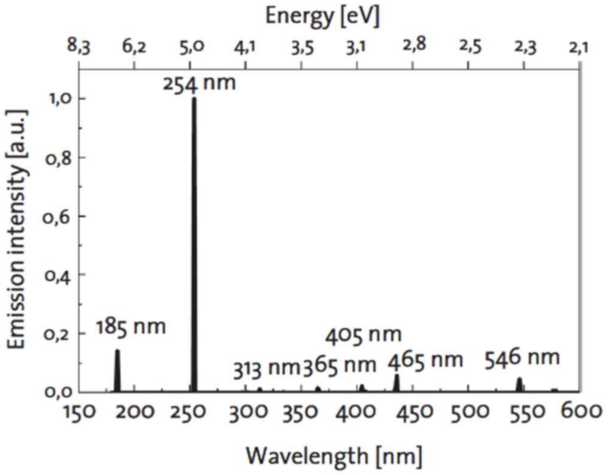

Figure S5. The spectrum of a low-pressure mercury lamp: It shows two peaks (185 and 254 nm), the major peak is 254 nm.
